# Factors impacting test-based management of suspected malaria among caregivers of febrile children and private medicine retailers within rural communities of Fanteakwa North District, Ghana

**DOI:** 10.1186/s12889-021-11960-w

**Published:** 2021-10-20

**Authors:** Olajoju Temidayo Soniran, Benjamin Abuaku, Abraham Anang, Patricia Opoku-Afriyie, Collins Ahorlu

**Affiliations:** 1 Department of Epidemiology, Noguchi Memorial Institute for Medical Research, College of Health Sciences, University of Ghana, Legon, Accra, Ghana; 2 Department of Science Laboratory Technology, Akanu Ibiam Federal Polytechnic, Unwana, Ebonyi State Nigeria; 3 Department of Parasitology, Noguchi Memorial Institute for Medical Research, College of Health Sciences, University of Ghana, Legon, Accra, Ghana

**Keywords:** Malaria, Test-based approach, Medicine retailers, Caregivers, Children

## Abstract

**Background:**

Prompt diagnosis and treatment prevents a mild case of malaria from developing into severe disease and death. Unfortunately, parasitological testing of febrile children is greater in the public and formal private sector than in the informal private sector in sub-Saharan Africa.

**Methods:**

A mixed method study was carried out to determine factors limiting test-based management of suspected malaria cases among caregivers of febrile children and Over-the-Counter medicine sellers (OTCMS) in eight rural communities in Ghana. Structured questionnaires were used to interview 254 adult caregivers. Fourteen in-depth interviews were conducted with OTCMS. The interviews were audio-recorded, transcribed verbatim, and analysed thematically.

**Results:**

The most frequently sought health providers by caregivers of febrile children in descending order were Community Health-Based Planning Services (CHPS) compounds; drug vendors; and OTCMS. Malaria parasitological testing rate of febrile children was highest (94.9%) at the CHPS compound and lowest (10.5%) at the OTCMS shops. Proportion of febrile children not subjected to malaria blood test is 28.3%. Among caregivers who did not ask for malaria blood test, 15.2% reported that healthcare provider did not offer a malaria blood test; 21.7% were financially handicapped to visit the Health Centre; and 63% lacked knowledge of malaria blood test and where to get it. From OTCMS point of view, clients’ inability to pay for malaria blood test, community perception that OTCMS are unqualified to perform malaria blood test, financial loss when unused RDT kits expires, clients’ demand for half dose of ACT, and activities of drug peddlers are factors limiting adherence to WHO recommended policy on testing before treating uncomplicated malaria cases.

**Conclusion:**

The study results suggest the need to implement community friendly interventions aimed at improving test-based management of suspected malaria in febrile children. These may include educating caregivers and community members on the need to test and confirm malaria in febrile children before treating them, and supply of subsidized RDT kits to OTCMS and re-training them to provide testing services to their clients. Further studies pertaining to influence of gender roles on healthcare seeking attitude for febrile children is also suggested.

**Supplementary Information:**

The online version contains supplementary material available at 10.1186/s12889-021-11960-w.

## Introduction

Malaria continues to claim more than 400,000 lives every year and the most vulnerable group are children under 5 years. Children accounted for 67% (272,000) of all malaria deaths worldwide in 2018. Access to care for febrile children remains too low and nearly 40% of such children in sub-Saharan Africa do not receive care from a trained medical provider [1]. The poorest of the poor in vulnerable communities in sub-Saharan Africa living in remote rural areas with limited access to health facilities suffer the most from malaria [2].

Currently, the World Health Organisation (WHO) recommends a confirmatory blood test for all suspected malaria cases and a prescription of artemisinin-based combination therapy (ACT) for those who test positive [3]. Prompt diagnosis and treatment prevents a mild case of malaria from developing into severe disease and death. The deployment of malaria rapid diagnostic tests (mRDTs) is a useful measure in the management of uncomplicated malaria particularly in highly endemic rural settings, where microscopy is a challenge [4].

In Ghana, private medicine retailers such as licensed Over the Counter Medicine Sellers (OTCMS) and pharmacies are often the most convenient sources of medicine to the majority of the population [5]. For the purpose of reducing obstacles in physical and geographical access to health care delivery to poorly served communities in Ghana, the Community-Based Health Planning and Services (CHPS) initiative became a national policy in 2000 [6], which led to the establishment of CHPS compounds as part of the public health system. In October 2003, the National Health Insurance Scheme (NHIS) was established under Act 650 with the primary goal of improving access to and quality of basic health care services through the establishment of mandatory district-level mutual health insurance schemes. The NHIS covers 95% of diseases including malaria and individuals exempted from paying premiums include children under 18 years, the aged (70 +) in the informal sector, and indigents [7]. Malaria case detection using mRDTs is successfully being implemented in public health facilities since 2010, but despite this, private medicine retailers are yet to be fully integrated into the implementation of test-based management of malaria [8, 9]. The test-based management of malaria became important because of the realization that the provision of artemisinin-based combination therapies (ACTs) and other antimalarials without parasitological confirmation frequently results in overtreatment of malaria, delays diagnosis and treatment of other causes of illness, and leads to wastage of the antimalarial drug [10]. This study was designed to explore factors limiting test-based management of suspected uncomplicated malaria among caregivers of children under 10 years and private medicine retailers in selected rural communities of Ghana.

## Methods

### Study area

**Fig. 1:**
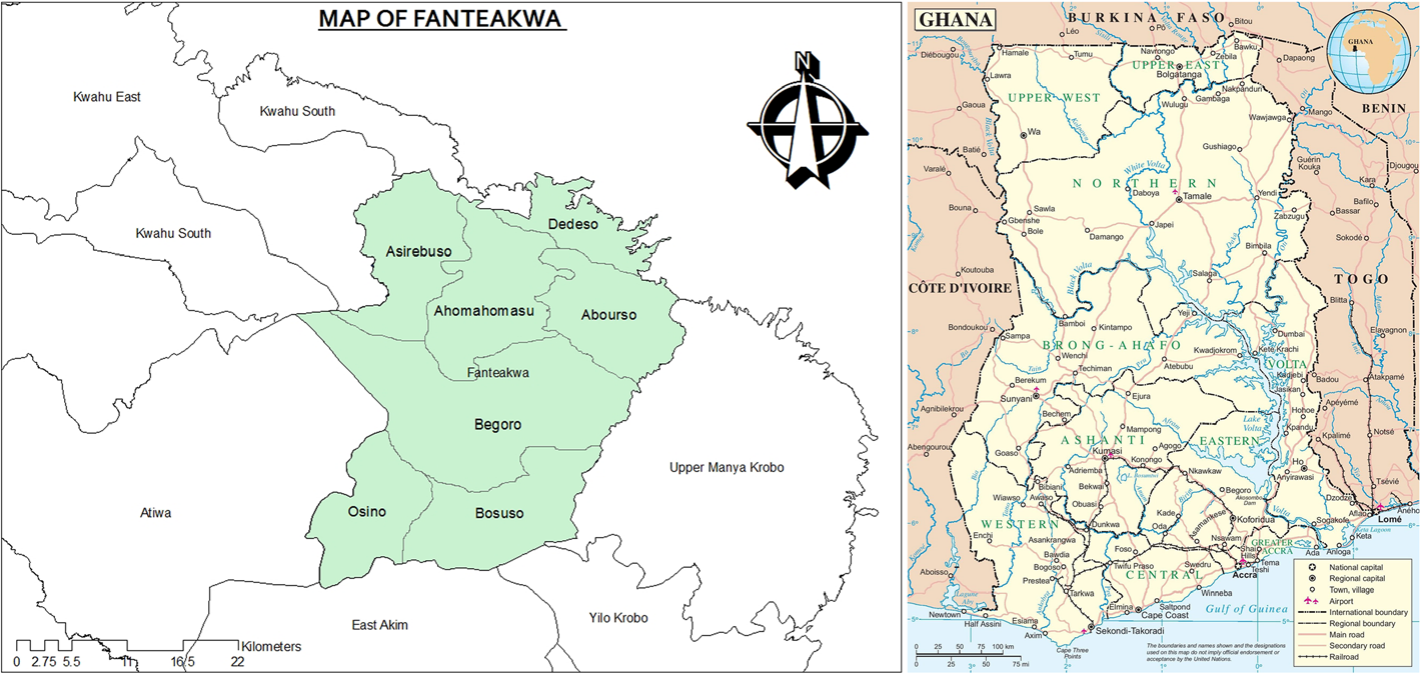
Map showing Fanteakwa North and Fanteakwa South districts (formerly one district – Fanteakwa)

This study was conducted in Fanteakwa North and Fanteakwa South districts (previously one district) in the eastern region of Ghana (Fig. 1). The study area has been described elsewhere [11].

A total of eight (8) communities (four in the intervention arm and four in the control arm) with at least one OTCMS were randomly selected from rural sub-districts within the two districts. Interventions deployed in the intervention arm were: facilitating acquisition of subsidized mRDT kits; training OTCMS on malaria diagnosis, treatment, and tracking of cases; quarterly supportive visits to OTCMS after training; community sensitization on malaria focusing on the T3 strategy; and introduction of malaria surveillance tool for use by OTCMS [11]. The selected communities were Ahomamu, Asirebuso, Dominase and Dedeso in Fanteakwa North district, and Ehiamakyene East, Ehiamakyene West, Bosuso, and Adjeikrom in Fanteakwa South district.

### Study design

This study, conducted between September and October 2019, was part of an ongoing implementation research aimed at improving the malaria test, treat, and track (T3) strategy [12] in the Fanteakwa North District (intervention arm) and Fanteakwa South District (control arm). The study was mixed in design (i.e quantitative and qualitative) using a household questionnaire survey targeting caregivers of children under 10 years in the intervention arm only and in-depth interviews (IDIs) of OTCMS in both the intervention and control arms.

Households used in the study were randomly selected from a sampling frame of households with children under 10 years old whilst OTCMS interviewed were from shops selected in both arms of the study. Sample size estimation has been described elsewhere [11].

Participants of the IDIs were either Owners/Operators of an OTCMS outlet or their Assistants in the eight (8) selected rural communities.

### Data collection

#### Community entry

The community entry was done by a team who visited the Chiefs and elders in the selected communities to explain the study objectives, and the need to sensitize the community members on mapping of households and ask for their supports and participation in the study. It was explained to them that participation was not compulsory but voluntary.

#### Data collection team

The quantitative study employed data collectors (school teachers) from each community who could conduct the interview in the local language (*Twi*) to ensure effective communication and accurate data capturing. The data collectors were trained on the purpose of the study and questionnaire administration.

Data collectors for the qualitative study included an experienced moderator and a note taker fluent in *Twi* language.

#### Household survey

A semi-structured questionnaire was developed, pre-tested for validity and administered by trained data collectors to respondents (caregivers/mothers of children under 10 years old). The questionnaire covered topics such as: Socio-demographics of respondent; knowledge of malaria and its transmission; history of fever among children under 10 years in the past 1 month; caregiver’s treatment seeking behavior; and insecticide treated bed net usage.

#### In-depth interviews with OTCMS

In-depth interviews (IDIs) were conducted using an interview guide developed in English and was pretested by direct translation into the local language by the moderator. The interviewer was a social scientist who had previous qualitative research experience. Major topics of interest covered in the guide included perceptions on symptoms of uncomplicated and severe malaria; knowledge of the policy on malaria testing, treatment and tracking (T3); any previous skill on the use of mRDT; and malaria management challenges faced in the past (Additional file 1). Interviews were conducted inside the OTCMS’ shops with each session lasting between 35 and 45 min. All interviews were audio-recorded and field notes taken to capture detail on the setting and non-verbal cues. In-depth interviews were mostly conducted in ‘*Twi*’ and translated into English during transcription.

In all, 14 in-depth interviews were conducted with seven OTCMSs (Owners/Operators) and seven OTCMS Assistants.

### Data analysis

Quantitative data were double entered and cleaned using Microsoft office Excel, 2013 version and exported to SPSS version 16.0 for analysis. Descriptive statistics were used to summarize all variables of interest. The dependent variable was the proportion of febrile children tested for malaria using mRDT while independent variables were demographic characteristics of study participants, their knowledge of malaria transmission and its cause, type of caregiver-child relationship, and sources of treatment. Chi-square test was used to determine association between the selected independent variables and number of febrile children tested before treatment (*p*-value ≤0.05 considered statistically significant). Transcripts of the qualitative data were thoroughly reviewed, emerging issues noted and codebooks developed. NVivo 12 (QSR International), a qualitative data analysis software program for coding, storage, indexing, and retrieval, was used to thematically analyze the transcripts [13]. Coding was carried out using predetermined themes based on the research questions and key areas of interest. Coding of all transcripts was carried out by a professional qualitative data analyst (Dr. Philip Teg-Nefaah Tabong) [14]. Results were presented in narratives based on the themes that emerged during the analysis.

## Results

### Socio-demographic characteristics of study OTCMS and caregivers

A total of 14 OTCMS were interviewed which included 7 shop owners and 7 attendant staff. Majority were males (85.71%); aged ≤50 years (57.14%); ≥10 years of experience (50%); married (57.14%); with Senior High School educational background (78.57%); Christians (92.86%); and practiced farming as an additional occupation (64.29%) (Table 1).

**Table 1:** Demographic characteristics of OTCMS service providers

Characteristics	n(%)	Attendant n(%)	Owner n(%)
	**14** (100)	**7** (50)	**7 (50)**
**Gender**			
Male	12 (85.71)	5 (71.43)	7 (100)
Female	2 (14.29)	2 (28.57)	0 (0)
**Age** (years)			
≤ 50	8 (57.14)	7 (100)	2 (28.57)
> 50	6 (42.86)	0 (0)	5 (71.43)
**Years of experience**			
< 10	7 (50)	7 (100)	0 (0)
≥ 10	7 (50)	0 (0)	7 (100)
**Marital Status**			
Single	6 (42.86)	6 (85.71)	0 (0)
Married	8 (57.14)	1 (14.29)	7 (100)
**Level of education**			
Junior High School	1 (7.14)	0 (0)	1 (14.29)
Senior high school	11 (78.57)	7 (100)	4 (57.14)
Tertiary	2 (14.29)	0 (0)	2 (28.57)
**Religion**			
Christianity	13 (92.86)	7 (100)	6 (85.71)
Islam	1 (7.14)	0 (0)	1 (14.29)
**Additional occupation**			
None	4 (28.57)	3 (42.86)	1 (14.29)
Farming/fishing	9 (64.29)	3 (42.86)	6 (85.71)
Petty trader	1 (7.14)	1 (14.29)	0 (0)

A total of 254 caregivers with children under 10 years old were interviewed. Majority were female (97.2%); aged 26–35 years (37.0%); married (80.3%); with Junior High School educational background (33.5%); Christians (91.3%); and farmers (49.6%) (Table 2).

**Table 2:** Demographic characteristics of caregivers of children under 10 years

Characteristics	n(%)	Asirebuso n(%)	Ahomamu n(%)	Dominase n(%)	Dedeso n(%)
	**254** (100)	**38** (14.96)	**91 (35.83)**	**72** (28.35)	**53** (20.87)
**Gender**					
Male	7 (2.76)	1 (2.63)	6 (6.59)	0 (0)	0 (0)
Female	247 (97.24)	37 (97.37)	85 (93.41)	72 (100)	53 (100)
**Age** (years)					
16–25	52 (20.47)	3 (7.89)	23 (25.27)	12 (16.67)	14 (26.42)
26–35	94 (37.01)	16 (42.11)	35 (38.46)	22 (30.56)	21 (39.62)
36–45	66 (26.98)	11 (28.95)	25 (27.47)	18 (25.00)	12 (22.64)
46–55	27 (10.63)	4 (10.53)	7 (7.69)	12 (16.67)	4 (7.55)
> 55	11 (4.33)	2 (5.26)	1 (1.10)	7 (9.72)	1 (1.89)
unknown	4 (1.57)	2 (5.26)	0 (0)	1 (1.39)	1 (1.89)
**Marital Status**					
Single	32 (12.59)	4 (10.53)	17 (18.68)	5 (6.94)	6 (11.32)
Married	204 (80.31)	33 (86.84)	71 (78.02)	57 (79.17)	43 (81.13)
Separated	6 (2.36)	0 (0)	1 (1.1)	3 (4.17)	2 (3.77)
Widowed	10 (3.94)	0 (0)	2 (2.20)	6 (8.33)	2 (3.77)
No response	2 (0.79)	1 (2.63)	0 (0)	1 (1.39)	0 (0)
**Level of education**					
No education	75 (29.53)	14 (36.84)	21 (23.08)	19 (26.39)	21 (39.62)
Primary school	70 (27.56)	10 (26.32)	24 (26.37)	28 (38.89)	8 (15.09)
Junior High School	85 (33.46)	11 (28.95)	32 (35.16)	21 (29.17)	21 (39.62)
Senior high school	15 (5.91)	2 (5.26)	10 (10.99)	1 (1.39)	2 (3.77)
Tertiary	7 (2.76)	0 (0)	4 (4.40)	2 (2.78)	1 (1.89)
No response	2 (0.79)	1 (2.63)	0 (0)	1 (1.39)	0 (0)
**Religion**					
None	3 (1.18)	1 (2.63)	2 (2.20)	0 (0)	0 (0)
Christianity	232 (91.34)	32 (84.21)	87 (95.60)	70 (97.22)	43 (81.13)
Islam	16 (6.30)	4 (10.53)	2 (2.20)	0 (0)	10 (18.87)
Others	1 (0.39)	0 (0)	0 (0)	1 (1.39)	0 (0)
No response	2 (0.79)	1 (2.63)	0 (0)	1 (1.39)	0 (0)
**Primary occupation**					
Unemployed	27 (10.62)	0 (0)	17 (18.68)	3 (4.17)	7 (13.21)
Farming/fishing	126 (49.61)	23 (60.53)	42 (46.15)	39 (54.17)	22 (41.51)
Petty trader	66 (25.98)	12 (31.58)	16 (17.58)	23 (31.94)	15 (28.30)
Civil servant	7 (2.76)	0 (0)	3 (3.30)	2 (2.78)	2 (3.77)
Artisan	16 (6.30)	0 (0)	12 (13.19)	1 (1.39)	3 (5.66)
others	10 (3.94)	2 (5.26)	1 (1.10)	3 (4.17)	4 (7.55)
No response	2 (0.79)	1 (2.63)	0 (0)	1 (1.39)	0 (0)

The caregivers sampled had a total of 492 children under their care. Majority of the children were male (54.1%); aged ≤5 years (55.89%); and direct biological children of the caregivers interviewed (83.7%) (Table 3).

**Table 3:** Demographic characteristics of children under 10 years

	n(%)	Asirebuso n(%)	Ahomamu n(%)	Dominase n(%)	Dedeso n(%)
**Characteristics**	**492 (100)**	**69 (14.02)**	**153 (31.10)**	**150 (30.49)**	**120 (24.39)**
**Gender**					
Male	266 (54.07)	41 (59.42)	83 (54.25)	82 (54.67)	60 (50)
Female	226 (45.93)	28 (40.58)	70 (45.75)	68 (45.33)	60 (50)
**Age (years)**					
≤ 5	275 (55.89)	43 (62.32)	87 (56.86)	73 (48.67)	72 (60)
> 5	217 (44.11)	26 (37.68)	66 (43.14)	77 (51.33)	48 (40)
**Caregiver-child relationship**					
Son/daughter	412 (83.74)	59 (85.51)	143 (93.46)	111 (74)	99 (82.50)
grandchild	58 (11.79)	10 (14.49)	6 (3.92)	33 (22)	9 (7.50)
Niece/nephew	20 (4.07)	0 (0)	3 (1.96)	5 (3.33)	12 (10)
Adopted/step child	1 (0.20)	0 (0)	1 (0.65)	0 (0)	0 (0)
Not related	1 (0.20)	0 (0)	0 (0)	1 (0.67)	0 (0)

### Knowledge of malaria

#### OTCMS’ knowledge of malaria

Service providers (OTCMS) were unanimous in indicating that malaria is caused and transmitted by the bite of the mosquito. Majority of them believe that the organism that causes malaria is deposited into the blood of the victim upon a mosquito bite. Two participants in this study described the cause of malaria as follows:

*“It is the same mosquito. If it bites someone and goes on to bite another, it will be spreading malaria. Malaria is not contagious. It is the mosquito that carries it around”* (Agyeikrom)

*“When it bites one person, with the blood still in the mouth, it will bite another person and transfer it to him”* (Ahomamon 4)

Service providers also mentioned activities that predispose people to malaria. Some activities identified were poor sanitation practices, indiscriminate disposal of empty canned tins and refusal to use bed nets. Interviewees indicated that indiscriminate disposal of used containers served as breeding grounds for mosquitoes that transmit malaria. These were identified by two participants as follows:

*“If stagnant water piles up in the containers we leave around. It breeds mosquitoes. Also, if we do not clean our gutters it becomes a dwelling place for mosquitoes, and they will cause the malaria”* (Agyeikrom)

*There are so many causes of malaria. Normally the mosquito lives in the chocked gutters and they mostly causes malaria. Chocked gutters and places with overgrown bushes breed mosquito, which comes out at night to bite us and cause malaria”* (Ahomamon 4)

These qualitative findings are similar to the majority stand of quantitative findings among caregivers of children under 10 years old.

#### Caregivers’ knowledge of malaria

Caregivers’ knowledge of malaria was assessed in terms of cause and transmission. Majority of caregivers (81.5%) mentioned mosquitoes as cause of malaria whilst only 3.5% mentioned “malaria germ” as cause. A mosquito bite, as mode of transmission was mentioned by majority of the caregivers (74%) (Table 4).

**Table 4:** Caregiver’s knowledge of malaria

Characteristics	n(%)	Asirebuso n(%)	Ahomamu n(%)	Dominase n(%)	Dedeso n(%)
	**254** (100)	**38** (14.96)	**91 (35.83)**	**72** (28.35)	**53** (20.87)
**Perceived cause of malaria**					
Malaria germ	9 (3.54)	0 (0)	0 (0)	6 (8.33)	3 (5.66)
Mosquito	207 (81.50)	22 (57.89)	82 (90.11)	62 (86.11)	41 (77.36)
others	33 (12.99)	15 (39.47)	7 (7.69)	2 (2.78)	9 (16.98)
Don’t know	5 (1.97)	1 (2.63)	2 (2.20)	2 (2.78)	0.(0)
**Perceived malaria transmission**					
Mosquito bite	189 (74.41)	28 (73.68)	79 (86.81)	43 (59.72)	39 (73.58)
Walking in the sun	24 (9.45)	1 (2.63)	4 (4.40)	17 (23.61)	2 (3.77)
Eating oily foods	9 (3.54)	2 (5.26)	1 (1.10)	4 (5.56)	2 (3.77)
Spiritual	1 (0.39)	0 (0)	0 (0)	0 (0)	1 (1.89)
others	31 (12.20)	7 (18.42)	7 (7.69)	8 (11.11)	9 (16.98)

### Management of malaria

#### OTCMS management of malaria

There was consensus that OTCMS manage malaria at the community level.

Service providers revealed that clients often present with signs and symptoms that are suggestive of malaria to their drugstore. The common signs and symptoms mentioned were fever, vomiting, body pains, feeling cold, and loss of appetite as illustrated by a quote from one of the study participants:

“*If they complain of high temperature and vomiting; if their urine is deep yellow then it’s probably malaria*” (Ehiamenkyene 1)

Interviewees also indicated that apart from the above signs and symptoms, some clients present with yellowish eyes, urine and palms. These signs according to participants are often suggestive of severe malaria. This was identified by two participants as follows:

*“….Yes, sometimes you can see from the person’s eyes. It becomes very yellowish and his palms becomes pale, with such manifestations you can say that condition is serious”* (Agyeikrom)

*“Sometimes it will make you have a high temperature and a severe headache, yellowish eyes which means it is severe”* {Ahomamu 1)

When clients report to their shops, OTCMS will ask the person about complains and the duration of the condition. This is used to know the severity of the client’s condition and determine if they can manage the condition on their own or refer to the clinic or hospital. Malaria that is deemed to be serious are referred to the clinic or hospital.

*“Some people have had the disease for a long time without treatment. In such cases I refer them to the hospital. But some people say it just started, so I treat those people”* {Ahomamu 1)

According to participants, clients are often given a painkiller, antimalarial and multivitamin syrup referred to as ‘blood tonic’. However, the type of medication and quantity is determined by the client’s ability to pay. Participants revealed that sometime clients are unable to afford all the medication, in such a case they are given the quantity and number of medication they can afford:

*“You know, I have to give them malaria medicine, Paracetamol and blood tonic. But some people can’t afford all of them, so I take the blood tonic out”* (Ahomamu 4)

*“First aid, I can give them paracetamol & Malafan (Sulfadoxine pyremethamine). If it is an elderly person, from 15 years above I give paracetamol & Malafan and multivitamin tablets before directing them to go to the hospital to check whether it is just malaria or something else*” (Ahomamu 2)

Also clients whose conditions do not improve after treatment are referred to the health facility. A participant shared his experience as follows:

*“When I give them the medicine and the condition does not get better, I refer the person to the clinic for further management”* (Agyeikrom)

These qualitative findings on management of malaria based solely on clinical symptoms support the minority position of the quantitative findings as reported by caregivers that visited the OTCMS.

#### Caregivers’ management of malaria

**Fig. 2:**
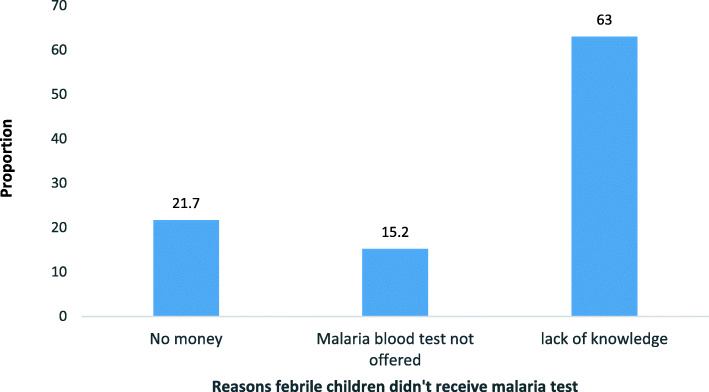
Caregivers' reasons for presumptive treatment of febrile children

The proportion of children reported to have had fever within 30 days prior to the survey was 30.9% (152/492). Majority of these febrile children (65.1% or 99/152) were reported to have been sent to a government health facility whilst 12.5% (19/152) were sent to OTCMS for care. There exists a significant relationship (*P* < 0.05) between conducting a malaria blood test and the provider of healthcare. A total of 94 of the children sent to a government health facility (95%; 95% CI: 88.1–98.1) were reported to have received a malaria test before treatment whilst only two (2) of the children sent to OTCMS (10.5%; 95% CI: 1.8–34.5) were reported to have received a malaria test before treatment. Children who received an ACT without a malaria test were 4(4.2%) at the government health facility and 8(80%) at the OTCMS (Table 5). Some of the reasons reported by caregivers whose children did not receive a malaria test were lack of money (21.7%), lack of knowledge on malaria blood test (63%) and malaria blood test not offered by healthcare provider (15.2%) (Fig. 2).

**Table 5:** Diagnosis and treatment of febrile children

Treatment		Sources of treatment and diagnosis							
		Caregiver		Drug vendors		OTCMS		CHPS compound	
	n(%)	Tested	Not tested	Tested	Not tested	Tested	Not tested	Tested	Not tested
**Herb**	**5** (3.3)	1 (20)	4 (80)	0 (0)	0 (0)	0 (0)	0 (0)	0 (0)	0 (0)
**ACT**	**127** (83.6)	1 (33.3)	2 (66.7)	10 (52.6)	9 (47.6)	2 (20)	8 (80)	91 (95.8)	4 (4.2)
**Antibiotics**	**9** (5.9)	0 (0)	1 (100)	1 (100)	0 (0)	0 (0)	4 (100)	3 (100)	0 (0)
**Others**	**11** (7.2)	0 (0)	4 (100)	0 (0)	1 (100)	0 (0)	5 (100)	0 (0)	1 (100)
**Total**		2 (15.4)	11 (84.6)	11 (52.4)	10 (47.6)	2 (10.5)	17 (89.5)	94 (94.9)	5 (5.1)
**Grand Total**	**152** (100)	**13** (8.6)		**21** (13.8)		**19** (12.5)		**99** (65.1)	

*OTCMS* over-the-counter medicine sellers, *CHPS* Community Health-Based Planning Services, *ACT* Artemisinin-based Combination Therapy

### Knowledge of OTCMS on test, treat and track (T3) malaria strategy

Most of the participants claimed that they were aware of the test, treat and track strategy, but their understanding revealed they lacked knowledge of the ‘track’ component of the T3. Participants were of the view that the purpose of ‘test’ is to know whether the client was really suffering from malaria before treating the condition. The following illustrate these points:

*“Test means that you need to find something. As I was saying, I tell people to go to the hospital to go and do a lab test to see exactly what is wrong with them. And “treat” refers to the treatment that is given to them. But I don’t understand the track”* (Agyeikrom)

*“The first word is Test. That means you have to test a person who comes to your shop with malaria, to see if it is malaria or not*” (Ahomamu 4)

*“….After the person comes to complain, firstly you need to test the person with RDT kit. That is what we use here because this place is not a lab where we can use machines to test. If you test the blood and read the results as positive you then give the drugs e.g. AA (Artesunate Amodiaquine) or AL (Artemether Lumefantrine). There are other drugs that accompany either the AA or AL…” (*Bosuso 2)

### Knowledge and training of OTCMS on malaria rapid diagnostic test (mRDT) kit usage

All participants in this study indicated they were aware of mRDT and its usage for diagnosing malaria before treatment. They described the process as pricking the finger of the client who report with signs and symptom suggestive of malaria, taking a drop of blood into the test kit and adding a buffer. Some of the participants are also aware of how to interpret test result. Some participants described how to use mRDT kit for diagnosis of malaria as follows:

*“It (mRDT) is used to check malaria. After removing the slip there is a small needle used to prick the person before putting it (blood) on the kit. It must be diluted with water (buffer) before putting it on the kit. It will show if the person has malaria”* (Ahomamu 2)

*“The test tube is in an envelope kind of thing. You open it and then use the needle to prick the person’s finger and when the blood comes out, there is something we use to collect the blood and put it on the tube. The blood will flow to one side of the device and will show a red sign. If the red sign appears twice the person is positive. If it appears once the person is negative”* (Ahomamu 3)

*“You prick the person’s hand with a needle. You wear gloves and squeeze a little. Blood will appear then you take it and put it on a tube and it will show. Lines appear. It might be one or two. Two is positive and one is negative” (*Ahomamu 1)

Some participants indicated they had received training on the use of mRDT to test for malaria. This training was organized by Pharmacy council mostly at the inception of the mRDT programme. Some participants shared their views as follows:

*“I remember we went for a training program organized by the pharmacy council. That is where I heard those words when they were teaching about malaria. But it’s been a long time so I have forgotten”* (Ehiamankyene 1)

*“I had my training in Tema where I used to be before coming here. Every year the pharmacy council trains us on new products that comes into market. So, we were trained on the usage of the test kit at its inception stage. That is during the time it was first introduced*” (Bosuso 2)

### Challenges faced by OTCMS on effective management of uncomplicated malaria cases

#### Test before treatment of malaria

The cost of the mRDT device according to participants is between GHS4-GHS5. However, clients are sometimes unable to afford the test. Hence they are sometimes compelled to treat without the test. Some participants shared their experience with clients as follows:

*“We have the malaria test which we use, but now it is costly so the ordinary people cannot buy at ¢5.00 before paying for the drug dispensed. So, if you people can help us with it (RDT) so that we test them for free, it will be good”* (Asirebuso 1)

*“But sometimes the person can insist on only the medicine; with that you cannot force the person for an RDT test. It can be due to financial problem*” (Bosuso 2)

There was consensus among participants that majority of the clients who report to their shops and are requested to do the rapid diagnostic test before treatment decline to do the test. According to participants, this decline is mostly due to poverty and lack of knowledge on importance of test before treatment. This is illustrated as:

*“I get about 10 people complaining of malaria on daily basis but only about 3 will agree to the test”* (Agyeikrom)

*“My challenge has to do with the fact that some people refuse the test. They will tell you that before the era of the test, they were still taking antimalarials, so just give them the drugs. I have realised that it has to do with financial difficulty. I believe that if they had the money they will insist on the testing in addition to the drugs. Also, most of the elderly are not educated on testing before treatment. You will talk at length yet they won't listen to you”* (Bosuso 2)

Interviewees therefore suggested government to supply OTCMS with the mRDT kits free of charge to enable them comply with the testing before treatment. This according to interviewees could help improve testing before treating suspected cases of malaria that report to their shops. The following illustrate this point:

*“The government should try and supply free test kits to the drug stores….because some clients are not able to afford the cost which is about GHS4*” (Ahomamu 3)

Apart from the cost which emerged as major barrier to the use of mRDT, some social barriers were also identified by participants. Some participants were of the view that clients sometimes resist the test because of the notion that OTCMS operators and their attendants are not trained medical personnel. Hence they lacked the expertise to correctly conduct the test and interpret the results as follows:

*“As I said initially, when you do the test here, they will think you are turning the place into a clinic, so I haven’t gone to buy some of the test kits. A while ago they said we should start selling those things. It was said at a meeting. But matters of this village differ from matters of the town. Even if they come and you want to test them, they will say you are not a doctor. So, as you people are coming to educate us (community), maybe next time we try to test them, they will understand”* (Asirebuso)

Clients’ belief in presumptive treatment of malaria based on signs and symptoms of malaria also emerged as barrier to testing before treatment as follows:

*“Just like how the young man who came to buy malaria drugs (a gentleman visited the shop for some painkillers), I asked what was wrong with him and he said he had a headache, he urinates yellow and he feels weak. So with him, I can tell him I will test him to confirm if its malaria. Other people also say they feel its malaria so give them medication. With that I’ll still say I want to test but others still refuse”* (Bosuso 1)

A participant also indicated that low patronage of the mRDT kits by their clients often lead to expiring of the product which has cost implications for shop owners. As a result many OTCMS will not stock mRDT as follows:

*“You know it has an expiry date, so most of the time they expire because the people can’t afford it. Then I have to throw it away. You see, if you always buy it only to throw it away due to expiry then that’s not good. It is also sold in full box not half that is the problem. So, if the cases come frequently, then I will go to buy. Sometimes I also go to borrow some few from the clinic”* (Dominase Quarters).

#### Treatment of suspected malaria cases

Majority of the participants also expressed challenge in the prescription of antimalarial medicine to their clients. Clients do demand to buy half dose of ACT because some can’t afford to buy the full dose at that point in time.

“…*Some people also complain of not having enough money to buy the full dosage of medicine (ACT) and may decide to buy a portion of it”* (Ehiamankyene 1)

“*Yes it happens. So they tell you to give them half so that when they are done with the half, they come and get the other half. Then I explain that the dosage is for 3 days so if it’s a child from 9 to 12 years, they take less depending on their weight. So with such kids half can be their dosage but with adults who take 4, they need to take the whole dose which is 24 tablets or else you wouldn’t fully recover. And after a month or two, there will be malaria present again*…….. *Some people will say they know all that but they insist on the half because they will come later”* (Bosuso 1)

*“Some people do not have enough money to buy the full dose of the medicine like the amodiaquine. They want you to cut a share for them according to the money they have. They won’t buy if you don’t divide it as they want. At times, looking at the condition of the person, you are forced to give them what they want even though you know it is not the right way of treating malaria”* (Asirebuso 1)

“*Some people don’t have enough money to buy the medicine. So, they can’t buy the full dosage. For example, AL. It’s 24. They sometimes ask for 12.”* (Ahomamu 1)

When asked if the clients that purchased half dose do come back to buy the remaining dose as promised, participants stated that some fail to come back especially when they feel relieved of symptoms. This was illustrated as:

“*Not all of them. Some say they are fine, others come and say they are feeling the same thing”*(Bosuso 1)

Participants complained that demand for half-dose of ACT by clients can be traced to the activities of drug peddlers. According to them, drug peddlers sabotage their efforts at maintaining the ethics of medical practice as stated by the Pharmacy Council. This is illustrated as:

“*We also have many drug peddlers around. Instead of giving the full dose or course, they can cut only 4 out of the 24 tablets. They are the cause of this habit. The government should do something about it. There are many of them in the village and Begoro here. On market days, they go around doing business in the hot sun. The drugs should be stored under a particular temperature, but they are selling under the sun. So, if you don’t sell the full box of drugs to the person, they will buy the divided one from the drug peddlers.”* (Ahomamu 5)

In some instance clients visit the hospital to have the test conducted but for some reason could not get the medicine. They therefore report to the OTCMS shop for the medicine. Participants shared their experience as follows:

*“Sometimes they come with a small plaster on their hand showing that they have been to the lab, and was confirmed that it is malaria, but they didn’t have money to buy the drugs there”* (Agyeikrom)

*“Some people go to the hospital to check if it’s malaria. They will be given medicine but if it is still positive after a while, they come to check for other alternatives”* (Ahomamu 1)

#### Perception of OTCMS on solution to challenges

The OTCMS unanimously suggested that government’s intervention is needed to address their challenges in the management of malaria and other illness within the community. The following illustrates their points:

*“The test kit should also be made available for us to buy and the price should come down for everyone to afford it. If the price is down (less expensive) the people will not hesitate to pay to be tested. Sometimes you don't even get it to buy”* (Bosuso 2)

“*The government should try and supply free test kits to the drug stores*” (Ahomamu 3).

“*Reducing the price of the medicine, so they (clients) can buy the right dosage”* (Ahomamu 1).

*“We also have many drug peddlers around. Instead of giving the full dose or course, they can cut only 4 out of the 24 tablets. They are the cause of this habit. The government should do something about it”* (Ahomamu 5).

*“The people should be educated on how to take malaria drugs. They see me to be a local guy and I am close to them (native of the community), so they won’t listen to me. But when people like you (Noguchi) comes to educate them, they will listen”* (Asirebuso 1).

## Discussion

Prompt diagnosis and treatment of confirmed positive cases is key to preventing a mild case of malaria from developing into severe malaria and death particularly in highly endemic rural settings [4]. In Ghana, test-based management of uncomplicated malaria has been scaled up in public health facilities but OTCMS are yet to be fully integrated into its implementation. Understanding factors responsible for delay in prompt diagnosis of malaria among caregivers and licensed OTCMS, who are the first point of call for majority of suspected malaria cases [15–17], is key to enhancing test-based management of malaria in rural communities.

Findings reported in this study show that OTCMS and caregivers have convergent views on the cause of malaria and its transmission. The reason is not far-fetched as OTCMS receive annual training from the Pharmacy council on recommended protocols in the management of uncomplicated malaria according to the national policy [18]. Caregivers of children are also educated on malaria in health talks by trained health workers during their antenatal visits at government approved health facilities [19]. The OTCMS and caregivers know that mosquitoes transmit malaria from person to person but their level of awareness on the pathogen (*Plasmodium*) that causes malaria is low. Identifying signs and symptoms suggestive of malaria is key to the OTCMS’ ability to manage uncomplicated malaria successfully. Fever and headache were frequently mentioned by OTCMS as part of the signs and symptoms of malaria which are in line with the National Malaria Control Programme policy on management of malaria [20].

Generally, clients’ inability to afford cost of a malaria blood test is a challenge for the OTCMS’ compliance with test-based management of malaria. Findings from this study showed that OTCMS are able to make an informed decision on whether to attend to a sick client or not based on severity of clients’ condition. However, clients suspected to have uncomplicated malaria are not offered malaria blood test because according to OTCMS’ experience, clients are often unable to pay for both testing and medications. Caregivers’ report also confirmed that malaria tests are rarely offered during their visits to the OTCMS. According to the caregivers, lack of money and knowledge of malaria blood test are among the reasons why a malaria blood test is not requested from the OTCMS. This is consistent with findings of previous studies [21] and this has led to high use of ACT without a malaria blood test. A similar study in Kenya also identified cost of conducting a malaria test as one of the barriers to implementation of mRDTs in retail outlets [22]. Prescription of ACT to unconfirmed malaria cases often lead to wastage of ACTs and may result to early development of drug resistance [10, 21]. Furthermore, the OTCMS prescribe some low cost medications often referred to as ‘first aid’ which may include *sulfadoxine pyremethamine (SP)-* an antimalarial drug meant for intermittent preventive treatment of malaria in pregnancy. Hence, over-prescription of SP at the community level by OTCMS may jeopardize its intended use under the national policy. On the other hand, government health facilities act as a facilitator of test-based management of malaria in the communities as reported by caregivers. At the CHPS compound (government facility), malaria RDT test is free for caregivers registered under the National Health Insurance Scheme (NHIS), but caregivers not registered under the scheme are required to pay between GHc3 - GHc5 (0.51USD – 0.86USD) for a malaria test (unpublished observation by Soniran O.T.). This finding supports previous documentation that parasitological testing of febrile children is greater in the public sector than in the informal private sector in sub-Saharan Africa [1].

In addition to the issue of affordability, OTCMS’ limited knowledge of the ‘track’ component of the T3 malaria strategy is of serious concern. It may be that there is little or no enlightenment on T3 during the annual training by the Pharmacy council. The ‘track’ component of the T3 strategy is meant to enhance monitoring of each patient and surveillance of all malaria cases with the goal of eliminating malaria in endemic countries [12]. Without confirming the real status of a suspected malaria case through a malaria blood test, it is impossible to properly track the patient. Findings in this study shows that OTCMSs have basic knowledge on how to conduct a malaria test using the RDT device while a few of them explained the interpretation of mRDT result correctly. The skill was learnt at the training by the Pharmacy council.

In one of the communities, an OTCMS reported a social barrier that limited his compliance with test-based management of uncomplicated malaria. Clients resisted the malaria blood test because of the notion that OTCMSs were not trained medical personnel. Similar study in Uganda reported that retail drug shops were viewed with suspicion and assumed to often operate outside the law, a perception that can affect mRDT implementation [23]. Adequate community sensitization on malaria and training of OTCMS are examples of interventional tools which if implemented may correct this notion.

The issue of affordability of malaria test has also been linked to persistent presumptive treatment of malaria as reported by the OTCMSs and this limits compliance with test-based management of malaria. It is common practice that caregivers try home treatment of malaria using combination of herbs or medications that had proved effective based on previous experience as reported in a previous study [24], and they visit OTCMS to seek health care or visit a government facility if there is no improvement in the health of their wards. This presumptive approach supports the chances of a mild case of malaria developing into a severe case and also delay the diagnosis of other illnesses causing fever in children [11, 25].

Loss of capital invested in purchased mRDT kits that later expires due to low clients’ compliance to getting tested also discouraged an OTCMS from re-stocking his shop with new mRDT kits. In the market, mRDT kits are sold in packs of 25 pieces/pack. The OTCMS lamented that sometimes, not all the 25 pieces of mRDT kits are used before the expiry date which results in his loss.

Prescription dosage of antimalarial drugs as stipulated by the National Malaria Control Programme of Ghana and W.H.O directives is gradually being undermined at the OTCMS shops as clients demand for half dosage of ACT due to lack of money to purchase the full dosage. Unfortunately, some of the clients that purchased half dose never return to purchase the remaining dose as they feel that they are okay. But if they did return, they demand for another medication with the belief that the ‘half dose antimalarial’ wasn’t effective. According to the OTCMSs, in addition to Clients’ lack of money, the habit can be traced to the activities of drug peddlers who fulfill the demands of their patrons. They also claimed that most of the drug peddlers are either unlicensed OTCMS or foreigners from neighboring African countries, selling antimalarial medicine at market places and under poor storage conditions. It is therefore obvious that continued under-exposure of malaria parasites to antimalarial drugs poses the threat of early development of *Plasmodium falciparum* resistance to ACT derivative drugs.

When OTCMS’ view were sought on how to overcome these limitations, they all reiterated that government should help in addressing these problems. They suggested a number of solutions including government supplying mRDT kits free of charge to them, educating the communities on test-based management of malaria and recommended dosage of ACT, subsidizing the cost of ACT, and addressing the issue of drug peddlers. However, there is limited evidence on how to improve test-based management of malaria in the informal health sector. Improved diagnostic and treatment practices in the private informal sector has the potential of impacting positively on universal access to diagnosis and treatment for malaria, and realization of the Sustainable Development Goal (SDG3).

This study has some limitations that should be noted. First, the study team interviewed caregivers to obtain data on presumed fever cases at the household level. Second, data presented is based on participants’ self-reports, which may be associated with some desirable bias. Lastly, the study did not explore caregivers’ knowledge of malaria symptoms, gender roles and its association with health care seeking behavior.

In conclusion, this study has shown that test-based management malaria is constrained by several factors in the rural communities. Caregivers’ lack of knowledge on malaria test, persistent presumptive approach to treatment of malaria, lack of money, financial loss by OTCMS on expired mRDT kits and perception that OTCMS are not qualified to perform malaria test are limitations observed. Presumptive treatment, an outdated policy, is further complicated with clients demanding for half dose of malaria medicine and activities of drug peddlers. These practices if unchecked, affects the potency of the antimalarial drugs, results to treatment failure, and may lead to early development of drug resistant malaria parasites. Increasing sensitization on malaria may improve caregivers’ attitude to proper management of suspected malaria in febrile children.

## Supplementary Information

**Additional file 1.** In-depth interview Guide for OTCMS.

## Data Availability

The datasets supporting the conclusions of this article are available in the Department of Epidemiology, Noguchi Memorial Institute for Medical Research and can be made available through the corresponding author on reasonable request.
