# Supplementary material for: Factors impacting test-based management of suspected malaria among caregivers of febrile children and private medicine retailers within rural communities of Fanteakwa North District, Ghana
Source: BMC Public Health. 2021 Oct 20;21:1899. doi: 10.1186/s12889-021-11960-w (PMC8527756; doi:10.1186/s12889-021-11960-w)
Supplement: Supplementary file 1 — Additional file 1. In-depth interview Guide for OTCMS. [file 12889_2021_11960_MOESM1_ESM.docx]

**In-depth interview Guide for OTCMS**

Name of community: ……………………………………………………………..

**Biodata**

Name: ______________________________________________

Sex: ______________________

Age: ______________________

Religion: ______________________

Marital Status: ______________________

Highest level of education: ______________________

Additional occupation: ______________________

Shop location/address: ______________________

Phone number: ______________________

**Questions**

(1) What do you think is the cause of malaria? Probe for *Plasmodium* species if not mentioned.

(2) How is malaria transmitted from one person to another person? Probe for mosquitoes if not mentioned

(3) As a drug shop keeper, have you come across any particular group of persons complaining of fever or malaria that you don’t attend to? Probe: Who are this group- age, sex, and why they are not attended to.

(4) How many classes of malaria in terms of severity are you aware of? Probe for- uncomplicated and severe malaria?

(5) When identified, how do you differentiate between the two types of malaria? Probe: signs and symptoms for each of them.

(6) Have you heard of test, treat and track (T3) malaria initiative before? Probe: tell us what you know about it.

(7) Have you been trained in the past on how to do malaria blood test using RDT? (let them show by raising their hands for a head count). Probe: who conducted or organized the training; where was it organized and when (year or how long ago) was the training done.

(8) Do you perform malaria test at your shop? Probe: the reasons for the testing; the cost of the testing; ability of clients to pay for the cost of testing; and how many clients suspected of having malaria are tested in a day presently.

(9) What are some of the challenges you face while attending to your customers (i.e malaria clients) that you wish authorities should address? Probe for District, regional and national levels.
